# Elevated Pre- and Postoperative ROTEM™ Clot Lysis Indices Indicate Reduced Clot Retraction and Increased Mortality in Patients Undergoing Liver Transplantation

**DOI:** 10.3390/biomedicines10081975

**Published:** 2022-08-15

**Authors:** Matthias Hartmann, Benedikt Lorenz, Thorsten Brenner, Fuat H. Saner

**Affiliations:** 1 Department of Anesthesiology and Intensive Care Medicine, University Hospital Essen, University Duisburg-Essen, 45147 Essen, Germany; 2 Department of General-, Visceral- and Transplant Surgery, University Hospital Essen, University Duisburg-Essen, 45147 Essen, Germany

**Keywords:** ROTEM™, liver transplantation, clot lysis index, cytochalasin D, clot retraction, mortality

## Abstract

Background: The ROTEM™ clot lysis index, describing the decrease in firmness of a clot with time, predicts mortality in various settings. The variability of the clot lysis index in surgical procedures and the involved pathophysiological mechanisms are unknown. We therefore compared pre- and postoperative clot lysis indices in liver transplantation (LTX) procedures, determined the eventual association with mortality, and investigated the mechanisms underlying decreased clot lysis index using inhibitors of fibrinolysis and clot retraction, respectively. Methods: In this retrospective cohort study, data on pre- and post-transplant ROTEM™ findings as obtained with EXTEM (tissue factor activation), INTEM (intrinsic system activation), FIBTEM (extrinsic system activation and inhibition of clot retraction), APTEM (extrinsic system activation and fibrinolysis inhibition), conventional laboratory coagulation tests, blood loss, transfusion of blood products, and outcome were registered. Results: Pre-transplant clot lysis indices showed a broad distribution ranging from 75% to 99% independent of the activator used (EXTEM, INTEM). During the surgical procedure, median clot lysis index values markedly increased from 92% to 97% (EXTEM) and 93% to 98% (INTEM), respectively (*p* < 0.0001 each). Aprotinin had no effect on either pre- or postsurgical clot lysis indices. Inhibition of platelet clot retraction with cytochalasin D (FIBTEM) markedly increased the preoperative clot lysis index. High pre- and post-transplantation clot lysis indices were associated with increased mortality irrespective of the activator used (EXTEM, INTEM) and the inhibition of fibrinolysis (APTEM). Inhibition of clot retraction (FIBTEM) abolished the association of clot lysis index with mortality in both pre- and post-transplantation samples. Conclusion: Both pre- and postoperative ROTEM™ clot lysis indices predict mortality in patients following liver transplantation. Inhibitor experiments reveal that the clot lysis index is not an indicator of fibrinolysis, but indicates platelet clot retraction. The marked increase of clot lysis index during liver transplantation is caused by a decrease in clot retraction with eventual consequences for clot stability, retraction of wound margins, and reperfusion of vessels in case of thrombosis.

## 1. Introduction

Rotational thromboelastometry (ROTEM™) is widely used as a bed side assay to evaluate hemostasis in liver transplantation and other clinical settings and is used to detect fibrin polymerization disturbances [1,2,3,4,5]. Moreover, recent studies demonstrate that the ROTEM™ clot lysis index predicts bleeding and mortality in various settings including liver transplantation (LTX), sepsis, and trauma [6,7,8,9,10,11,12]. The ROTEM™ clot lysis index describes the decrease of the maximum firmness of a clot with time as the percentage between clot firmness after 60 min and maximum clot firmness. In the above-mentioned settings, a high ROTEM™ clot lysis index (which describes only a sparse decrease in maximum clot firmness over time) is associated with increased mortality. In LTX, categorization of ROTEM™ clot lysis index allowed to identify a low-risk group (clot lysis index < 85%) with a mortality of 3% and a high-risk group (clot lysis index > 95%) with a mortality of 25% in a recent study [6]. Similarly, 30-day sepsis mortality increased with an odds ratio of 85 in patients with a clot lysis index higher than the median at the onset of the disease [8].

The prognostic value of the ROTEM™ clot lysis index has been explained as a fibrinolytic system inhibition in many clinical settings including LTX, sepsis, and trauma. Accordingly, increased levels of plasminogen activator inhibitor I and thrombin activatable fibrinolysis inhibitor are thought to be the correlate of the findings achieved with viscoelastic methods [9,13]. Therefore, a high clot lysis index is termed “fibrinolytic shutdown”. However, recent findings from our group demonstrate that differences in clot lysis index cannot necessarily be ascribed to fibrinolysis [6].

While the effects of pre-transplantation ROTEM™ clot lysis index on mortality and other outcome measures has been demonstrated, it is not known whether the variable is constant in an individuum or might be affected by the transplantation procedure. Moreover, it is not known whether the post-transplantation clot lysis index predicts mortality as well. To evaluate the mechanism of the clot lysis index, we investigated whether a low clot lysis index can be explained by fibrinolysis or clot retraction using selective inhibitors.

## 2. Materials and Methods

### 2.1. Patient Data

After allowance of the ethics committee of the University Hospital Essen (09-4091) for the retrospective data analysis, data from 187 complete pre- and postoperative data sets were investigated in this retrospective study. Both conventional laboratory variables (aPTT, INR, fibrinogen, antithrombin, fibrinogen, and platelet count) and ROTEM™ coagulation variables (clot lysis index, clotting time, clot formation time, maximum clot firmness, and alpha angle) were determined at the beginning of LTX as well as at the end of the procedure. Moreover, patients’ age, sex, disease necessitating LTX, model of end stage liver disease score (MELD), blood loss, and coagulation factor substitution were recorded. For the generation of preoperative Kaplan–Meier curves, further 166 preoperative data sets were used.

### 2.2. Procedures

Anesthesia was performed as recently described, and was introduced with thiopental and maintained with isoflurane and fentanyl [14]. For facilitation of endotracheal intubation, rocuronium was used. A radial artery catheter, a central venous catheter, and a pulmonary artery catheter, as well as transesophageal echocardiography, were used for hemodynamic monitoring. A catheter was inserted in the femoral vein for the detection of eventual vena cava stenosis. Hypovolemia and anemia were treated with infusion of sodium chloride (0.9%) and red blood cell concentrates using a rapid infusion device (FMS-2000, Belmont Instruments Corporation, Billerica, MA, USA). Fibrinogen concentrate, prothrombin complex concentrates, and platelet concentrates were administered in case of coagulopathy and dosage was guided by ROTEM™, Coulter counter results, and occurrence of diffuse bleeding. In all patients without cancer, intraoperative cell-salvage was used. Surgery was performed with a vena cava replacement technique.

Rotational thromboelastometry (ROTEM™ Roteg 05 device, Werfen, Barcelona, Spain) and conventional laboratory values were used to determine the effect of LTX on clot lysis index. Four ROTEM™ tests were used to investigate the citrated whole blood: 1. EXTEM test (tissue factor for extrinsic system activation), 2. INTEM test (ellagic acid for intrinsic system activation), 3. APTEM test (tissue factor activation combined with aprotinin to inhibit fibrinolysis), and 4. FIBTEM test (tissue factor activation in presence of cytochalasin D to inhibit clot retraction). The ROTEM™ tests at the beginning and the end of the transplantation procedure were evaluated.

### 2.3. Statistics

SPSS Statistics (version 27, IBM, New York, NY, USA) was used for the evaluation of data. Figures were generated with SPSS Statistics and OriginPro (Originlab, Northampton, MA, USA). Mean and standard deviation, median and quartiles, box plots, pair plots, and Kaplan–Meier analyses were used to describe the data. For statistical analyses, Wilcoxon tests, linear regression analyses (Pearson), and 2-tailed significance analyses, as well as log-rank tests, were used, as appropriate.

## 3. Results

### 3.1. Patient Characteristics

The present study includes 187 adult patients, 51% of which were male. Median age was 51 years and median lab-MELD-score was 23. The 30-day mortality after LTX was 13.9% (*n* = 26). The diseases leading to LTX in this study are listed in Table 1. Causes for 30-day mortality were sepsis (*n* = 16), bleeding (*n* = 3), transplant failure (*n* = 2), cardiac events (*n* = 1), and other reasons (*n* = 4). Median as well as quartiles and range (given in brackets) of blood products applied in this series were as follows: red blood cell concentrates 3 U (quartiles 0–6 U; range 0–40 U), fibrinogen 2 g (0–4 g; 0–19 g), prothrombin complex concentrates 0 U (0–0 U; 0–5000 U), and platelet concentrates 0 U (0–1 U; 0–7 U).

### 3.2. Distribution of Pre-Transplantation Clot Lysis Index

**Figure 1:**
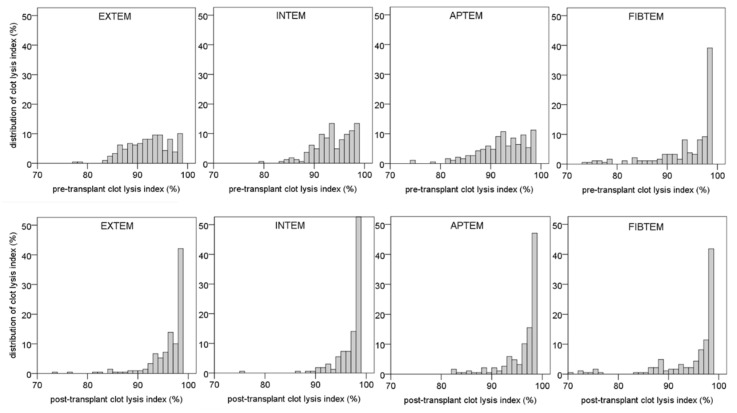
Distribution of clot lysis index obtained with ROTEM™ at the beginning (upper row) and at the end of liver transplantation (lower row) using four different assays (EXTEM: tissue factor activation, INTEM: ellagic acid activation; APTEM, tissue factor activation and fibrinolysis inhibition with aprotinin; FIBTEM: tissue factor activation and platelet clot retraction inhibition with cytochalasin D).

**Figure 2:**
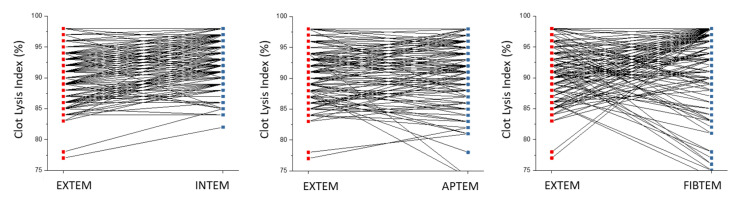
Pair plots showing the similarity between clot lysis index values obtained with EXTEM, INTEM, and APTEM. In contrast, inhibition of clot retraction with FIBTEM increased the clot lysis index in many cases.

The pre-transplant clot lysis index, as determined with rotational thromboelastometry, showed a broad distribution in the EXTEM, INTEM, and APTEM tests with a range from 75% to 99% (Figure 1). The pre-transplantation clot lysis indices determined with EXTEM, INTEM, and APTEM test were highly correlated (r = 0.7; *p* < 0.0001). Differences of clot lysis index values obtained with the EXTEM and INTEM tests were 1.6% ± 4.0% (mean and SD), demonstrating that the clot lysis index is not influenced by the activator used (extrinsic vs. intrinsic system). Moreover, there was no significant difference in the clot lysis index between the EXTEM and APTEM tests, which amounted to −0.1% ± 3.6% (mean and SD), thus indicating that differences in the clot lysis index between patients were not due to differences in fibrinolysis. Inhibition of platelet clot retraction with cytochalasin D (FIBTEM test) shifted the distribution of the presurgical clot lysis index to higher values in the post-transplantation phase (Figure 1). A comparison of individual pre-transplantation clot lysis index values in the EXTEM, INTEM, APTEM, and FIBTEM tests using pair plots are summarized in Figure 2.

### 3.3. Distribution of Post-Transplantation Clot Lysis Index

**Figure 3:**
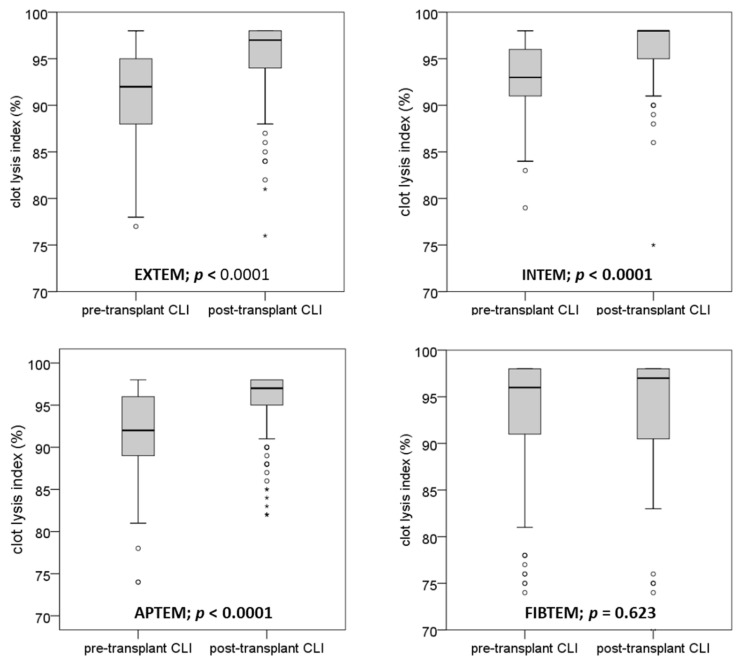
Boxplots depicting the median and quartiles of clot lysis index values obtained at the beginning and the end of liver transplantation. ROTEM™ tests were performed with extrinsic and intrinsic system activation (EXTEM, INTEM), as well as in presence and absence of a fibrinolysis inhibitor (APTEM) and a platelet clot retraction inhibitor (FIBTEM), respectively. The Wilcoxon test was used for statistical evaluation. Outliers are indicated by circles an asterisks.

**Figure 4:**
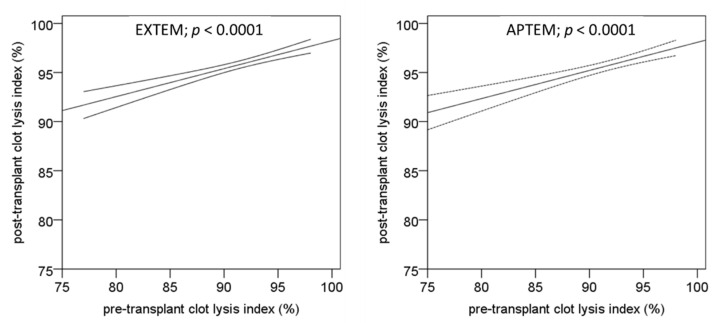
Linear correlation and confidence intervals (of the mean, 95%) of pre-transplant and post-transplant clot lysis index as obtained by rotational thromboelastometry. Whole blood samples were activated with tissue factor (EXTEM, left figure) and tissue factor combined with a fibrinolysis inhibitor (APTEM, right figure).

After the surgical procedure, distribution of clot lysis index was shifted to higher values in the EXTEM, INTEM, and APTEM tests (Figure 3). The median clot lysis index significantly increased in these tests within the transplantation procedure from median 92–93% to 97–98% (*p* < 0.0001 each). In contrast, the pre- and post-transplantation clot lysis indices in the FIBTEM test (means: 96% and 96%, respectively) were not different (*p* = 0.623). Linear regression analysis obtained from pre- and post-transplantation clot lysis index values in the absence or presence of aprotinin (EXTEM and APTEM tests) demonstrates particularly that the low clot lysis index values increased during surgery and that the fibrinolysis inhibitor did not have any effect (Figure 4).

### 3.4. Association of Clot Lysis Index at the Beginning and the End of Transplantation with Mortality

**Figure 5:**
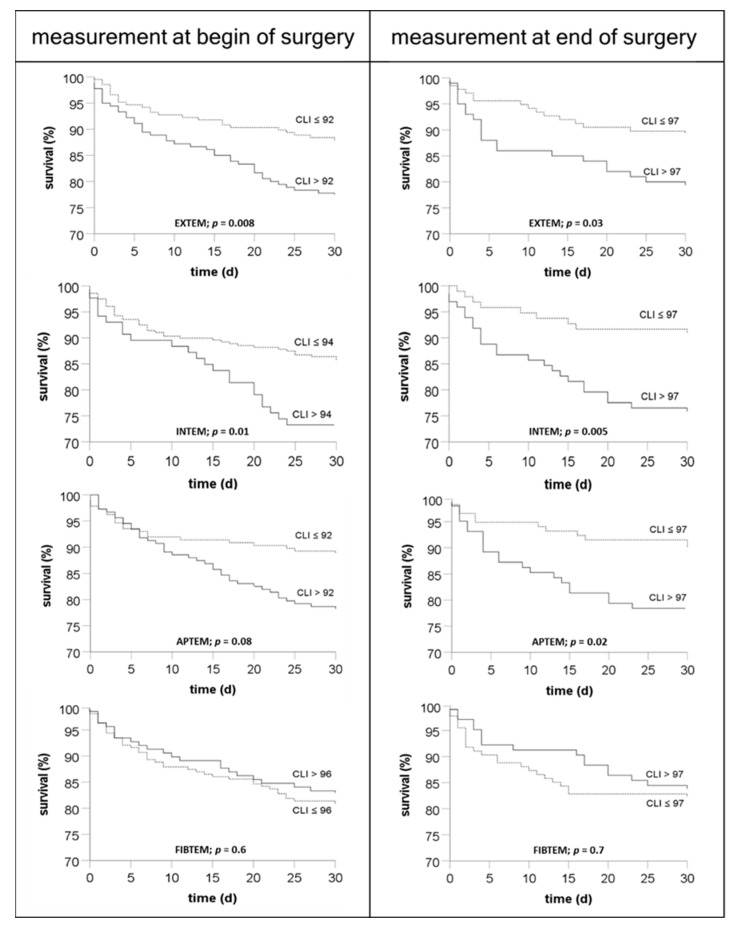
Association of ROTEM™ clot lysis indices obtained from patients at the beginning and the end of liver transplantation with 30-day-survival. Results demonstrate that a clot lysis index higher than the median in the EXTEM, INTEM, and APTEM test is associated with reduced survival. Inhibition of clot retraction using cytochalasin D in the FIBTEM test resulted in a loss of the association of clot lysis index with mortality. For statistical evaluation the log-rank test was used.

To estimate the association of clot lysis index with outcome, the survival of patients with a clot lysis index higher and lower than the median, respectively, were compared (Figure 5). The Kaplan–Meier curves, obtained from measurements at the beginning and the end of the transplantation showed a lower survival in the EXTEM, INTEM, and APTEM tests in patients with a clot lysis index higher than the median of the group. In contrast, inhibition of clot retraction using cytochalasin D in the FIBTEM test abolished the predictive value of the clot retraction index.

### 3.5. Correlation of Pre-Transplantation Clot Lysis Index with Other Laboratory Findings

Among conventional coagulation tests, INR (r = 0.321; *p* = 0.0002), activated partial thromboplastin time (r = 0.310; *p* = 0.0002), antithrombin (r = −0.180; *p* = 0.03), and platelet count (r = −0.207; *p* = 0.01) were correlated with pre-transplantation clot lysis index, while fibrinogen was not correlated (r = −0.150; *p* = 0.10). Moreover, pre-transplantation clot lysis index was correlated with the MELD-score (r = 0.323; *p* = 0.0001). Correlation of pre-transplantation clot lysis index with other ROTEM™ variables was low and not significant in most tests (Table 2).

### 3.6. Correlation of the Post-Transplant Clot Lysis Index with Blood Products

Linear correlation analysis excluded that red blood cells (r = 0.113; *p* = 0.178), fibrinogen-substitution (r = 0.112; *p* = 0.180), prothrombin complex concentrates (r = 0.117; *p* = 0.161), platelet concentrates (r = 0.123; *p* = 0.142), volume substitution (r = 0.031; *p* = 0.714), tranexamic acid (r = 0.076; *p* = 0.366), and duration of surgery (r = 0.056; *p* = 0.492) were correlated with the clot lysis index.

## 4. Discussion

The results of the present study demonstrate that ROTEM™ clot lysis index markedly increased during liver transplantation. This increase cannot be attributed to differences in the fibrinolytic system, the perioperative blood loss, or the substitution of blood products. However, inhibition of clot retraction by use of cytochalasin D demonstrated that inhibition of platelets’ contractile force development increases clot lysis index values. Moreover, comparison of the clot lysis index at the beginning and the end of surgery demonstrates a marked increase during the surgical procedure. Increased pre- and postoperative clot lysis index values were associated with decreased survival. This result is in good accordance with the findings in sepsis and trauma, and might suggest a modulation of platelets’ clot retraction caused by inflammatory events.

Effect of surgery on clot lysis index: While it is known, that the clot lysis index can serve as a biomarker for outcome in sepsis, trauma, and LTX, it is unknown whether a certain value is a constitutive characteristic of a patient or whether the variable can change during surgical procedures [6,7,8,10]. The present study is the first demonstrating directly that changes in clot lysis index can occur within a few hours of surgery.

Fibrinolysis shutdown and clot lysis index: The common interpretation for the increase in the ROTEM™ clot lysis index (which is equivalent to increases in the thrombelastography clot lysis variable) is the so called “fibrinolytic shutdown”, supposed to be caused through increases in plasminogen activator inhibitor I and thrombin activatable fibrinolysis inhibitor [11]. However, the present study falsifies the hypothesis of the clot lysis index as a measure of fibrinolysis shutdown in liver transplantation, as the inhibition of fibrinolysis with aprotinin did not affect clot lysis index.

Association of clot lysis index with mortality: Both pre- and postoperative clot lysis indices were associated with mortality. This finding was not dependent on extrinsic and intrinsic system activation using the EXTEM and INTEM test, respectively. Moreover, inhibition of fibrinolysis using aprotinin had no effect on the association of clot lysis index and mortality. However, inhibition of clot retraction using cytochalasin D abolished the association both pre- and postoperatively.

Associations of laboratory variables with clot lysis index: The results of the present study demonstrate that the pre-transplantation clot lysis index is positively associated with the MELD-score, activated prothrombin time, and INR, and negatively correlated with antithrombin levels and platelet count. Interestingly, the clot lysis index was not associated with other ROTEM™ variables. These findings demonstrate that clot lysis index alterations are more pronounced in those patients with decreased liver function and thus might be a biomarker of disease severity.

Mechanism of alterations in clot lysis index in liver disease and transplantation: Concerning the mechanism responsible for an altered clot lysis index, the effect of platelet inhibition in the FIBTEM test is remarkable and suggests an involvement of platelets. Indeed, clot retraction, which is specifically inhibited by cytochalasin D in the FIBTEM test, could represent the mechanism responsible for the differences in clot lysis indices. Accordingly, it has been hypothesized that viscoelastic methods are sensitive for clot retraction [15]. However, viscoelastic methods seem neither to be sensitive nor specific for clot retraction and standard tests for clot retraction must be done to prove the hypothesis.

Platelets are not only critically involved in primary hemostasis, but are also immune cells. Platelets are affected by inflammatory mediators, can release many mediators from their granules, can induce the contraction and stabilization of clots, and are involved in wound healing [16,17,18,19]. Moreover, effects of various diseases on clot retraction have been demonstrated [16]. Thus, platelets might represent sensors for inflammation.

There are some limitations of the study. Due to the retrospective design, it is not possible to further evaluate the importance of mechanisms leading to the increase in the clot lysis index during surgery. Thus, further studies are necessary to evaluate the pathophysiological mechanisms involved.

Conclusions: The present study demonstrates that the clot lysis index, an indicator of 30-day mortality, increases during liver transplantation in a platelet-dependent way independent of fibrinolysis. Clot retraction is the most probable mechanism explaining the differences in clot retraction and the association with mortality.

## Figures and Tables

**Table 1:** Diseases leading to liver transplantation. Frequencies are given in absolute values and percentages.

Disease Leading to LTX	Cases	Percentage
alcohol	38	20.3
cholestatic diseases	24	12.8
hepatocellular carcinoma	32	17.1
hepatitis	23	12.3
re-transplant	14	7.5
inherited diseases	13	7.0
non-alcoholic steatohepatitis	12	6.4
acute liver failure	11	5.9
autoimmune diseases	7	3.7
biliary atresia	5	2.7
intoxication	3	1.6
tumor	3	1.6
cryptogenic	2	1.1

**Table 2:** Linear correlation of clot lysis index obtained with four ROTEM™ tests (EXTEM, INTEM, FIBTEM, APTEM) with the ROTEM™ variables CT, alpha angle, CFT, and MCF. Asterisks indicate significant correlations (*p* < 0.05).

ROTEM	EXTEM	INTEM	FIBTEM	APTEM
CT	0.133	0.089	0.02	0.005
Alpha	0.153 *	0.082	0.03	−0.022
CFT	−0.073	−0.235 *	0.148	−0.011
MCF	−0.202 *	−0.246 *	0.151	−0.049

## Data Availability

The data are available on reasonable request.
